# Rare Taxa and Stochastic Drift Drive the Microbiome Assembly of the Invasive Pest *Drosophila suzukii*, While Host Filtering Structures the Grape Sour Rot Community

**DOI:** 10.1007/s00248-026-02819-x

**Published:** 2026-06-26

**Authors:** Rishi Bhandari, Zoe Wills, Jackie Harris, Marnelle Salie, Nana Y. D. Ankrah, Adam Chun-Nin Wong, Katharine Eastman, Joseph Ringbauer, R. Keith Striegler, David S. Kang

**Affiliations:** 1 Biological Control of Insects Research Laboratory, United States Department of Agriculture, Agricultural Research Service, Columbia, MO USA; 2 E & J. Gallo Winery, Acampo, CA USA; 3 Biological Science Department, State University of New York Plattsburgh, Plattsburgh, NY USA; 4 Department of Entomology and Nematology, University of Florida, Gainesville, FL USA

**Keywords:** Community assembly, Stochastic processes, Rare taxa, Spotted wing drosophila

## Abstract

Understanding the processes that shape microbial community structure is a central challenge in ecology. The relative importance of deterministic and stochastic processes in driving the microbiome assembly and composition remains poorly understood across diverse animal and plant hosts. Here, we characterized the bacterial (16 S rRNA) and fungal (ITS rRNA) communities in invasive pest spotted wing Drosophila (SWD, *Drosophila suzukii*) and sour-rot-affected grapes from seven California vineyards using high-throughput sequencing. We found that while the bacterial microbiome of male and female SWD was compositionally similar, these communities were entirely distinct from those on sour rot-affected grapes, indicating strong host-specific filtering. SWD also harbored substantially higher microbial density and greater diversity compared to grapes. Ecological modeling revealed a fundamental divergence in assembly mechanisms: SWD communities were predominantly shaped by stochastic processes, with a neutral model explaining 40% of the variation. In contrast, sour-rot-affected grape communities were structured by a combination of stochastic and deterministic factors. Importantly, taxa that deviated from neutral predictions, thereby indicating selection, were primarily classified as rare or intermediate in abundance. This suggests that low-abundance taxa may act as keystone drivers of dysbiosis during rot development. These results highlight the differing assembly rules governing a vector-host system: the mobile vector acts as a stochastic reservoir, promoting pathogen dispersal, while the sessile host imposes strict environmental filtering. This underscores the critical role of rare taxa in shaping community structure and ecosystem stability in SWD and its host grapes.

**Supplementary Information:**

The online version contains supplementary material available at https://doi.org/10.1007/s00248-026-02819-x.

## Introduction

Host-microbe interactions are crucial for the survival, thriving, and dominance of microbial communities in their respective environmental niches. The assembly of the plant microbiome is influenced by a complex interplay of biotic and abiotic factors, including host genetics, climatic and edaphic conditions, pathogen invasion, insect activity, and others [1–3]. In the presence of biotic and abiotic stresses, plants assemble beneficial microbes to maximize their growth and survival [4, 5]. Similarly, insects are known to selectively recruit and maintain specific microorganisms under the pressure of natural selection, employing immune systems and providing specialized nutrients [6–8]. Notably, a strong microbial linkage between plants and insects is emerging, influencing host physiology and the overall health of ecosystems. However, the intricate nature of microbial interactions among different ecosystem components remains poorly understood.

The composition of the microbiota in plants and insects is influenced by a wide range of factors, including geographic location, pH, temperature, precipitation, nutrient availability, and host genotype [1, 9, 10]. However, the extent to which each factor influences microbial community composition and assembly varies significantly across host species. Two distinct processes (deterministic and stochastic) govern microbial community assembly, with their relative importance varying across systems and scales. Deterministic processes involve interactions between species, such as competition, predation, and symbiosis [11]. The stochastic process assumes equal fitness levels and shapes the community structure through random ecological drift, including births and deaths [12, 13]. Exploring the ecological patterns governing the assembly and co-occurrence of host-associated microbiomes, and the influence of environmental and host factors on these patterns, provides valuable insights into the mechanisms underlying microbial community assembly and composition. Understanding how microbial spatial patterns are controlled is a central yet poorly understood topic in microbial ecology [14]. Microbial communities often consist of abundant and rare taxa, each contributing unique functions to their hosts. Abundant taxa primarily drive ecosystem functions, while rare taxa provide structural and functional stability during stress, acting as ecological insurance [15–17]. These studies shed light on contrasting community patterns and processes in abundant and rare subcommunities, which are influenced by distinct environmental factors and play a crucial role in shaping community assembly processes [18].

Beyond assembly, research on microbial ecology has paid particular attention to how these communities change across space, uncovering a pattern known as distance decay. The distance-decay relationship indicates that microbial community similarity across samples declines as the geographic distance between them increases. This effect has been widely studied in bacterial and fungal communities across diverse environments, such as natural forests, grasslands, lake ecosystems, and agricultural areas [19–22]. Several factors contribute to this distance-decay relationship in the microbiome, including but not limited to environmental heterogeneity [23], dispersal limitation [24, 25], microbiota turnover [22, 26], and stochastic processes [27, 28]. Studying distance-decay patterns helps us understand how community similarity declines with spatial separation, allowing us to disentangle the relative influences of dispersal limitation, environmental filtering, and stochastic drift on microbial assembly.

Spotted wing Drosophila (SWD) (*Drosophila suzukii* Matsumura), a close relative of *D. melanogaster*, is an agricultural pest with a broad host range and can infest small, soft-skinned, and stone fruit [29]. SWD is presumed to be native to Asia, but the invasive pest was first reported in California in 2008, where it was observed infesting strawberries and cranberries [30]. Since its introduction to North America, SWD has rapidly spread, causing significant economic losses [31, 32]. Unlike its congeneric relatives, female SWD has a serrated ovipositor that can pierce the skin of healthy, soft-skinned fruits to lay eggs [33]. While doing so, adult SWD introduces various microorganisms, including yeasts and acetic acid bacteria, to the fruit, thereby affecting the fruit and the wine.

In grapes, both *D. melanogaster* and SWD promote the development of sour rot disease [34, 35]. Grape sour rot is a disease complex characterized by the acetic acid smell and browning of grape berries. It is caused by a community of acetic acid bacterial genera *Acetobacter* and *Gluconobacter*, and yeasts [35, 36]. The distinctive symptoms of the disease include pre-harvest decay accompanied by the presence of acetic acid. The acetic acid further attracts fruit flies, which helps spread sour rot both within and between clusters, as well as throughout the vineyard [34, 37]. These microbial communities, which are known to cause sour rot in crops, significantly affect the fitness and functions of SWD flies, including their development, behavior, lifespan, stress tolerance, fecundity, immunity, and resistance to various biotic and abiotic stresses [38–45]. Although the most abundant microorganisms in grape berries affected by sour rot and associated Drosophila flies have been well-documented, few studies have yet explored the assembly process of these microbial communities in both SWD and grapes. The roles of both abundant and rare microorganisms in this assembly process remain largely unexplored.

This study highlights the intricate interactions among microbiomes and insect vectors that contribute to the development of sour rot in grapes. By employing high-throughput sequencing of the 16 S rRNA and ITS rRNA genes, we investigated the microbial communities comprising bacteria and fungi in the fruit fly SWD and its host, grapes. Additionally, we categorized microbes into abundant and rare taxa to (1) compare their biodiversity and biogeographical patterns and (2) quantify the impacts of stochastic processes, which was crucial to unraveling the ecological processes and mechanisms responsible for maintaining the stability of this ecosystem. Disentangling the ecological processes underlying microbial assembly in this crop-pest system provides a deeper understanding of the dynamics within this agroecosystem and their implications for managing grape sour rot.

## Materials and Methods

### Sample Collection

Specimens of the Spotted Wing Drosophila (SWD) were collected from seven vineyard areas in California: Clarksburg, Walnut Grove, Woodbridge, Acampo, Clements, Victor, and Galt (Supplementary Data 1, Supplementary Fig. 1). The flies were collected using modified Biogents BG-Sentinel 2 traps (Biogents AG, Germany), commonly used for mosquito surveillance. Each trap was baited with a Trécé PHEROCON SWD broad-spectrum lure (Great Lakes IPM, Michigan, USA). The traps were powered by a 12-V battery pack, which powers a small fan that pulls and directs approaching flies into a collection bag. During each trapping period, traps were placed at least 100 m apart. Traps were operated for 24 h. The following morning, collected flies were removed from the traps and stored at 4 °C. The flies were then transported to the laboratory, sorted by sex, and identified under a stereomicroscope (Olympus SZX-12, Tokyo, Japan) using standard morphological identification keys. Our sampling covered four winegrape cultivars, Sauvignon Blanc, Chardonnay, Petite Sirah, and Pinot Noir, representing the species’ interaction with different grape cultivars. This experimental design helps us to investigate the influence of geographic location, fly sex, and grape cultivar on the microbial community composition within the SWD. In addition to the SWD specimens, grape samples were also collected from each location and cultivar.

### DNA Extraction

DNA was extracted from flies using the DNeasy Blood and Tissue Kit (Qiagen, Germany). Single fly samples were added to tubes containing 1.2 g of 0.1 mm silica beads (Qiagen, Germany) and subjected to bead beating for 1 min and 30 s at 3000 RPM using the Mini-Beadbeater-96 (BioSpec, Bartlesville, OK, USA). DNA extraction for the flies was performed following the manufacturer’s protocol, except that 25 µl of nuclease-free water was used for elution in the final step. To extract DNA from grapes, 5 g of grape samples were cut and sonicated for 15 min in phosphate-buffered saline solution (50 mM) supplemented with 0.02% Tween 20. The dislodged cells were pelleted and processed for DNA extraction. DNA was extracted using the Wizard Genomic DNA Purification Kit (Promega, Madison, WI, USA), according to the manufacturer’s guidelines. DNA quantification was performed using a Qubit 3.0 fluorometer (Thermo Fisher, Waltham, MA, USA) and a NanoDrop 2000 spectrophotometer (Thermo Scientific, Wilmington, DE, USA). The extracted DNA was stored at −80 °C until further processing. The samples were then sent to Novogene Inc. (California, USA) for amplicon sequencing. Among the samples sent for 16 S and ITS2 sequencing were 46 DNA samples (split evenly between 23 male and 23 female specimens) and 16 grape samples (Supplementary Data 1, Supplementary Fig. 1).

### PCR Amplification and Sequencing

Both the flies and grape DNA were subjected to PCR amplification targeting the V3–V4 regions of the 16 S rRNA gene using primers 341 F (CCTAYGGGRBGCASCAG) and 806R (GGACTACNNGGGTATCTAAT) for bacteria [46] and the ITS2 region using primers ITS3-2024 F (GCATCGATGAAGAACGCAGC) and ITS4-2409R (TCCTCCGCTTATTGATATGC) for fungi [47]. Library construction and sequencing were performed on the Illumina NovaSeq, generating 250-bp paired-end reads.

### Sequence Data Processing

Raw paired-end FASTQ files obtained from sequencing were subjected to primer sequences removal using Cutadapt [48] prior to downstream processing. Following primer removal, sequence data processing was performed using the high-resolution Divisive Amplicon Denoising Algorithm 2 (DADA2) package (v1.32) [49] in R [50]. The read quality was inspected with “*plotQualityProfile*” and the reads were filtered using the “*filterAndTrim*” function. Sequences containing any ambiguous bases were discarded (maxN = 0). Individual reads are truncated at the first nucleotide base with a Phred quality score lower than 2 (truncQ = 2). Additionally, a stringent quality filter was applied, discarding reads if the expected number of errors exceeded 2 for both forward and reverse reads (maxEE = c (2, 2)). Following filtration, sequencing error rates were estimated independently for the forward and reverse reads using the “*learnErrors*” function with default parameters. This step generates a parametric error model from the dataset to distinguish true biological sequence variants from sequencing errors prior to dereplication and denoising. Reads were subsequently dereplicated, denoised, merged, and assembled into a sequence table. Chimeric sequences were detected and removed using the consensus method with the “*removeBimeraDenovo”* function in DADA2. The taxonomy assignment was performed with the “*assignTaxonomy*” function using the SILVA database (silva_nr99_v138.1) [51] for 16 S rRNA gene sequences and the UNITE database (sh_general_release_04.04.2024) [52] for ITS region sequences. We constructed a phylogenetic tree from the denoised ASVs by first aligning the sequences and generating an initial neighbor-joining tree using the DECIPHER package (v3.0.0[53]. This preliminary tree and alignment were then used in phangorn (v2.12.1) [54] to infer a maximum-likelihood phylogeny under a Generalized Time-Reversible (GTR) model with gamma-distributed rate heterogeneity, and the resulting tree was midpoint-rooted. The ASV table, phylogenetic tree, and taxonomic placement were joined to a phyloseq object in R using PhyloSeq package (v1.48) [55]. For the bacterial community analysis, the Phyloseq object was strictly filtered to exclude unassigned kingdoms and phyla, as well as chloroplast and mitochondrial sequences. ASVs belonging to the endosymbiont *Wolbachia* were also removed and retained for separate downstream analysis. Similarly, ASVs assigned to non-eukaryotic taxa and to unassigned kingdoms and phyla were removed from fungal community analyses.

PhyloSeq objects obtained for bacterial and eukaryotic data were filtered to exclude ASVs with fewer than 10 counts, and these accounted for less than 0.001% of the total sampling depth. A total of 62 samples (46 flies and 16 grapes) were collected from four grape cultivars at seven sites (Supplementary Data 1, Supplementary Fig. 1). After quality filtering, denoising, and chimera removal, a total of 2,033,994 paired-read bacterial sequences were obtained from the 62 samples. The sequences ranged from 5,908 to 57,696, with an average of 32,806 ± 16,538 reads per sample (Supplementary Data 2A). After removing unassigned taxa (domain-level assignment) and singletons, chloroplasts, mitochondria, and the endosymbiont genus *Wolbachia*, and ASVs with a total count less than 10 and present in less than two samples, 134 unique ASVs belonging to 34 families and 46 bacterial genera were recovered (Supplementary Data 3A).

Out of the total 62 samples, 24 SWD samples, and none of the grape samples amplified for the ITS gene for accurate fungal composition analysis. This could be due to the low microbial biomass in these samples. Therefore, in this study, we were unable to compare the fungal microbial communities between SWD and grape samples. Of the 22 SWD samples, three from the cultivar Walnut Grove and Sauvignon Blanc were removed because they lacked replicates. The remaining 19 samples yielded 889,226 paired fungal reads, with an average of 46,796 ± 10,997 reads per sample (Supplementary Data 2B). These reads corresponded to 56 fungal ASVs belonging to 11 families and 12 genera (Supplementary Data 3B). The rarefaction curves of bacteria (Supplementary Fig. 2A) and fungal richness (Supplementary Fig. 2B) (observed ASVs) were plotted against sequencing depth, indicating that all samples had sufficient reads to capture most of the bacterial and fungal community diversity, allowing further analysis to proceed.

Chao1 [56] and Shannon diversity [57] indices were estimated from the phyloseq object using the “*estimate_richness*” function in the phyloSeq package. To evaluate the effects of host type (male flies, female flies, and grapes), sampling location, and grape cultivars on microbial richness and diversity, Generalized Linear Mixed-Effects Models (GLMMs) were constructed using the glmmTMB package (v1.1.14) [58] to accommodate the specific non-normal error distributions inherent to ecological diversity metrics. Chao1 richness, representing overdispersed count data, was modeled using a Negative Binomial distribution (nbinom2 family with a log link function) after rounding estimates to the nearest integer. Shannon diversity, which is continuous and strictly positive, was modeled using a Gamma distribution (with a log link function).

For bacterial communities, the main GLMMs included host type, grape cultivar, and their interaction as fixed effects, while sampling location was modeled as a random intercept to account for spatial autocorrelation. To explicitly isolate the impact of SWD sex, independent GLMMs were run exclusively on the SWD samples, evaluating sex as a fixed effect and location as a random effect. To quantify the influence of spatial variance in these models, the Adjusted Intraclass Correlation Coefficient (ICC) was calculated using the performance package (v0.16) [59], representing the proportion of unexplained variance attributable to sampling location. Due to a restricted sample size (*N* = 19), fitting spatial covariates for the fungal communities resulted in rank-deficient design matrices and aliased coefficients. Consequently, the sampling location random effect was explicitly excluded from these models. The fungal models were simplified to test the influence of host type and cultivar.

For all GLMMs, model assumptions were validated using simulation-based diagnostics via the DHARMa package (v0.4.7) [60]. The significance of fixed effects was determined using Type III Wald chi-square tests to ensure the independent assessment of main effects in the presence of interactions. Post-hoc pairwise comparisons were conducted using estimated marginal means using the emmeans package (v1.10.7) [61] with a Benjamini-Hochberg correction.

The Phyloseq object’s read counts were then normalized using cumulative sum scaling with the metagenomeSeq package (v1.46) [62]. This normalization step explicitly corrects for varying sequencing depths across samples, preventing library size disparities from confounding distance metrics. Microbial beta diversity was assessed by computing Bray-Curtis distance matrices on the normalized read counts. The ordinations for beta-diversity between treatments were estimated using a Non-Metric Multidimensional Scaling (NMDS) analysis based on Bray-Curtis dissimilarity matrices, as implemented in the *“plot_ordination”* function in the PhyloSeq package. The effect of host types, sampling locations, and grape cultivars on bacterial community dissimilarity was tested using permutational multivariate analysis of variance (PERMANOVA) with the “*adonis2*” function in the vegan package (v2.6–10) [63]. First, the effects of host type, grape cultivar, and their interaction were tested, with permutations constrained within sampling location using the strata argument (strata = location). This constrained permutation structure was used to account for non-independence among samples collected from the same location. To further identify differences among treatments, a pairwise multi-level comparison was performed using the “*pairwise.adonis*” function in the pairwiseAdonis package (v0.4.1) [64] using Benjamini-Hochberg adjusted p-values. Similarly, the effect of sampling location on bacterial community composition was tested using a separate PERMANOVA model with location as the explanatory variable. Pairwise PERMANOVA comparisons were used to identify differences among locations. Due to the restricted fungal sample size, spatial variables were excluded. PERMANOVA was conducted to test the main effects of host and cultivar on differences in the fungal community. Multivariate dispersion among the treatments (deviation from centroids) was assessed using the functions “*betadisper*” and “*permutest*” from the vegan package. Feature-level analysis was performed to identify taxa with differential abundance between treatments using Linear models for differential abundance analysis (LinDA) implemented through the “*generate_taxa_test_single*” function in the MicrobiomeStat package (v1.2.1) [65]. LinDA utilizes traditional linear models with center log-ratio (CLR)- transformed abundance data and a bias-correction procedure to account for the compositional structure of microbiome data, employing regularization techniques to enhance the robustness and accuracy of differential abundance detection. For the bacterial dataset, independent models were constructed to evaluate two distinct biological questions while controlling environmental heterogeneity. First, to assess taxonomic enrichment driven by the insect host environment, models tested the main effect of the broad host category (SWD vs. Grapes), with grape samples as the reference. Second, to evaluate sex-specific insect microbiomes, an independent model was constructed using only SWD samples. To rigorously control confounding variables and prevent spurious taxonomic associations, both bacterial models included the cultivar and the sampling location as fixed covariates with the function “*adj.vars*”. Consistent with the overarching statistical framework, the spatial location covariate was excluded from the fungal LinDA models to avoid rank deficiency given sample size limitations. For all tests, statistical significance was evaluated using the Benjamini-Hochberg procedure to control the false discovery rate (FDR), with differentially abundant taxa defined by an adjusted *p*-value < 0.05.

Total microbial communities and microbial communities across each host type were visualized by UpSetR package (v1.4.0) [66]. The core microbial community of different host types was investigated using the microbiome package (v1.26.0) [67]. We defined a core community as the ASVs common across the various host types, using different prevalence thresholds and based on shared community composition. This definition used a minimum prevalence of 0.70 and a detection threshold of 0.01, given the greater variation among wild communities, especially with smaller sample sizes. Results were plotted as Venn diagrams to visualize exclusive and shared core members using the VennDiagram package (v1.7.3) [68].

### Analysis of Microbiota Density

We define microbiota density as the total DNA extracted from each sample (µg) per mg of fresh tissue [69]. Prior to analysis, the distributions of the continuous variables (body weight and microbiota density) were assessed for normality using the Shapiro-Wilk test. Because the body weight data significantly deviated from normality, differences between adult males and females were analyzed using a two-sample Wilcoxon rank-sum test.

Microbiota density was log10-transformed prior to downstream analyses to reduce skewness. To evaluate if fly sex influenced microbial density independently of body size, an analysis of covariance (ANCOVA) was performed on the insect samples. This linear model evaluated log10-transformed microbial density against sex, log10-transformed body weight, and their interaction.

To compare the microbial density between the fly and grape samples, a non-parametric Kruskal-Wallis rank-sum test was utilized across all three groups. Significant main effects were subsequently analyzed using post hoc pairwise Wilcoxon rank-sum tests, with p-values adjusted using the Benjamini-Hochberg procedure to control the false discovery rate. Statistical significance was established at an alpha level of *p* < 0.05. Visualizations were generated using the ggplot2 package to create hybrid box-and-flat-violin plots.

### Analysis of Abundant and Rare Taxa

We employed the ulrb (Unsupervised Learning-based Definition of the Rare Biosphere) package (v0.1.6) [70] to classify microbial ASVs into abundance categories (e.g., rare, intermediate, or abundant) across each sample. Unlike traditional approaches that depend on arbitrary thresholds for relative abundance, ulrb employs unsupervised machine learning using the partitioning around medoids (PAM) algorithm. This robust k-medoids clustering technique groups ASVs into abundance categories by minimizing within-cluster dissimilarity. The optimal number of clusters (e.g., three categories: rare, intermediate, and abundant) was determined using the average Silhouette score, a statistical measure of clustering quality that evaluates both intra-cluster cohesion and inter-cluster separation. We then classified each ASV as abundant or rare across the population based on its prevalence: an ASV was considered abundant or rare if it exhibited that status in more than 50% of the samples in which it was present. Additionally, we identified the intermediate taxa as conditionally rare or conditionally abundant ASVs, which are typically rare under certain conditions but occasionally or periodically reach high abundance, and vice versa.

### Compositional and Phylogenetic Distance-Decay Analyses

Distance–decay relationships were evaluated to understand whether microbial community dissimilarity increased with geographic distance among sampling locations. The geographic distance (kilometers) between sample locations was calculated using the Haversine formula [71]. Subsequently, the resulting distance matrices were analyzed with the Mantel test, employing Spearman’s rank correlation and permuting 999 times using the vegan package. Furthermore, regression lines were included in distance–decay plots to visualize the direction of the relationship. Additionally, phylogenetic distance-decay analyses were performed using the beta net relatedness index (betaNRI), which provides a standardized measure of the mean phylogenetic distance to the nearest taxon within the community using the “*comdistinct*” function in the Picante package (v1.8.2) [72].

### Community Assembly Process Analysis

To assess the potential significance of stochastic processes in community assembly, we employed a neutral community model to predict the correlation between the detection frequency of amplicon sequence variants (ASVs) and their relative abundance across the broader metacommunity. By fitting our data to a mechanistic neutral model [13], we aimed to establish the relationship between the prevalence of microbial species and their average relative abundance within those communities. This model enables us to estimate the proportion of microbial species in a dataset that exhibits an abundance-prevalence relationship consistent with the neutral model. In other words, well-predicted ASVs (those with abundance and prevalence within the model’s 95% confidence limits) are likely influenced by stochastic factors such as dispersal and ecological drift. Conversely, ASVs that deviate from the 95% confidence interval (CI) of the neutral model predictions are more likely to be affected by deterministic factors, such as host selection [73, 74].

We then calculated the beta nearest-taxon index (βNTI) to quantify phylogenetic turnover between microbial communities relative to the null model. βNTI represents the number of standard deviations of the beta mean nearest taxon distance from the mean of the null distribution [75, 76]. A βNTI greater than 2 and less than − 2 indicates that the community is shaped by deterministic processes (positive values: variable selection, negative values: homogeneous selection). When βNTI falls between − 2 and + 2, it indicates that stochastic processes have guided community succession [76, 77]. The null model analyses were conducted using the “*cal_ses_betamntd*”, “*cal_rcbray*”, and “*trans_nullmode*l*”* function in microeco package (v1.12.0) [78]. Together, the neutral model quantified the contribution of stochastic processes based on patterns of abundance. At the same time, βNTI incorporated phylogenetic turnover to distinguish deterministic selection from stochastic variation, providing complementary insights into the community assembly mechanism.

### Co-occurrence Network Analysis

Microbial co-occurrence networks were inferred in R using NetCoMi package (v1.1.0) [79], with association strength between the taxa estimated via the SPRING (Semi-Parametric Rank-based approach for INference in Graphical model) method [80]. Briefly, ASVs were agglomerated to the genus level, and taxa were filtered to reduce sparsity using the detection threshold of 0.0001 and a prevalence threshold of 10%. The co-occurrence network was first inferred using the “*netConstruct*” function, and the properties were evaluated using the “*netAnalyze*” function in NetCoMi. The modularity was assessed using the “*cluster_fast_greedy*” algorithm in the NetCoMi package. To identify hub taxa that play crucial roles in the networks, we set a threshold of 0.95 for eigenvector centrality. Finally, we compared the hub taxa across the treatment groups using the Jaccard similarity index. Networks across host type were compared using the “*netCompare*” function in NetCoMi with 1,000 permutations.

## Results

### The Natural Variation of Body Weight and Microbiota Density in Flies is Influenced by Host Sex

**Fig. 1:**
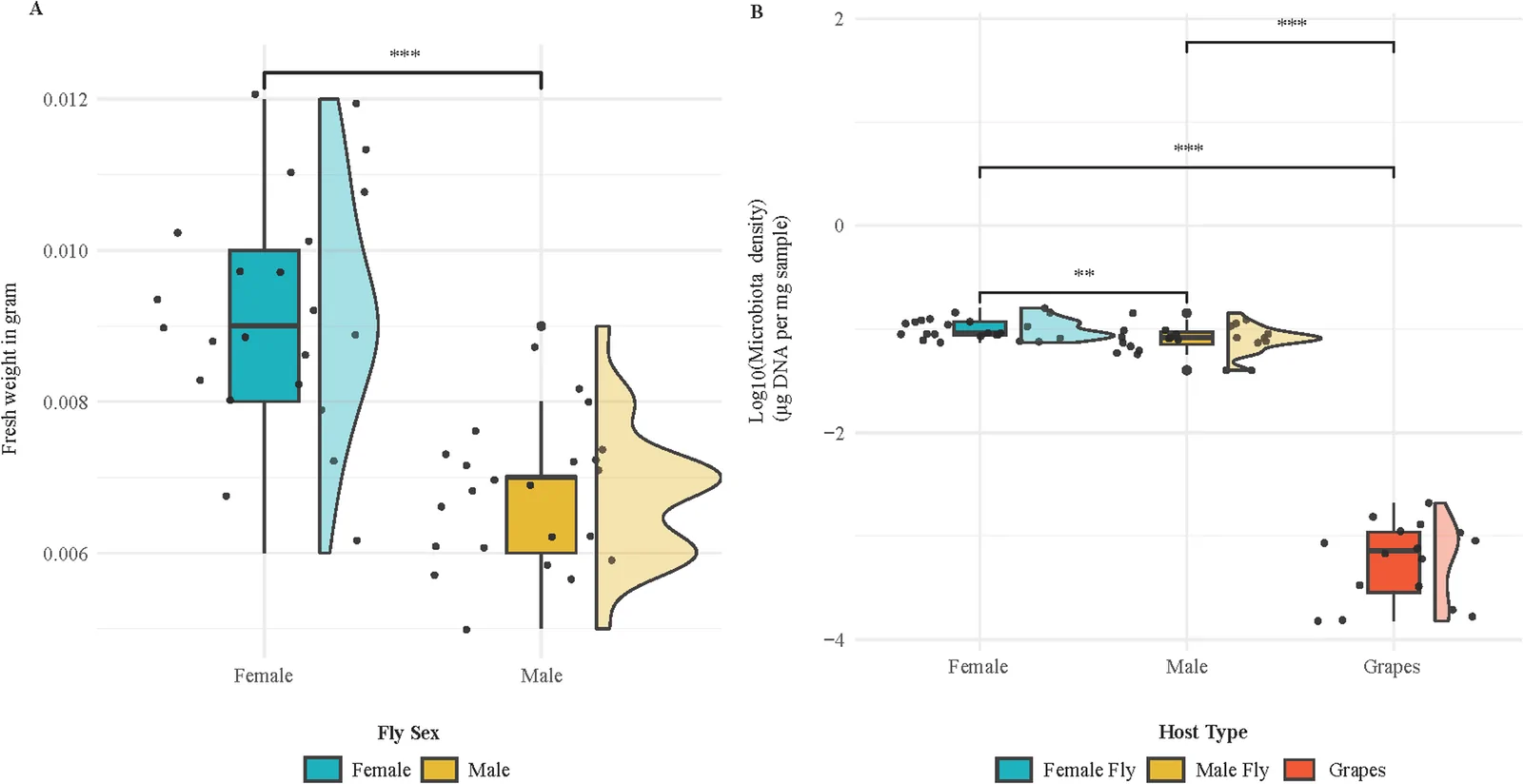
Sex and host factors influence natural variation in fly weight and microbiota density across flies and grapes. Mean fresh weight (g) across male and female flies (**A**). A comparison of the log_10_-transformed microbiota density (micrograms of DNA per milligram of sample) between male, female flies, and grape samples (**B**). Box plots show the median and interquartile range, with whiskers extending to values within 1.5 times the interquartile range. Individual points represent observed samples, and the density/violin shape illustrates the overall distribution of observations within each group. Microbial density was log₁₀-transformed prior to plotting and statistical analysis. Statistical comparisons were performed using the Wilcoxon rank-sum test, and the significance between groups is denoted by asterisks: * *p* < 0.05; *p* < 0.01; *** *p* < 0.001

The mean individual fresh weights for male flies were 6.78 ± 0.19 mg, while female flies had a mean weight of 9.22 ± 0.33 mg. We observed significant differences in fresh body weight between the two sexes (Wilcoxon rank-sum test, W = 479.5, *p* < 0.001) (Fig. 1A), indicating that males are significantly lighter (approximately 1.4 times) than females.

To evaluate whether the absolute quantities of microbiota differ between grapes and flies, we evaluated DNA-based microbiota density, expressed as micrograms of microbial DNA per milligram of sample. Within the insect samples, microbiota density was significantly influenced by fly sex (ANCOVA main effect of sex: F_1,42_ = 9.27, *p* = 0.004) but was independent of the body weight (ANCOVA main effect of body weight: F_1,42_ = 2.52, *p* = 0.120) (Fig. 1B). Specifically, female flies harbored a higher raw microbiota density (0.102 ± 0.005) compared to males (0.082 ± 0.005). Additionally, we observed a significant difference in microbiota density between grapes and both male and female flies (Kruskal-Wallis test, χ2 = 38.96, df = 2, *p* < 0.001). Post-hoc pairwise comparisons confirmed that microbiota density in grapes was significantly lower than in both male and female flies (Wilcoxon rank-sum tests with Benjamini-Hochberg correction, *p* < 0.001 for both comparisons) (Fig. 1B). These findings suggest that sex-specific factors influence both body weight and microbial composition. Furthermore, microbiota density in grapes appears to be significantly lower compared to that of flies.

### Location has a Substantial Influence on Microbial Community Diversity and Richness in SWD and Grapes

**Fig. 2:**
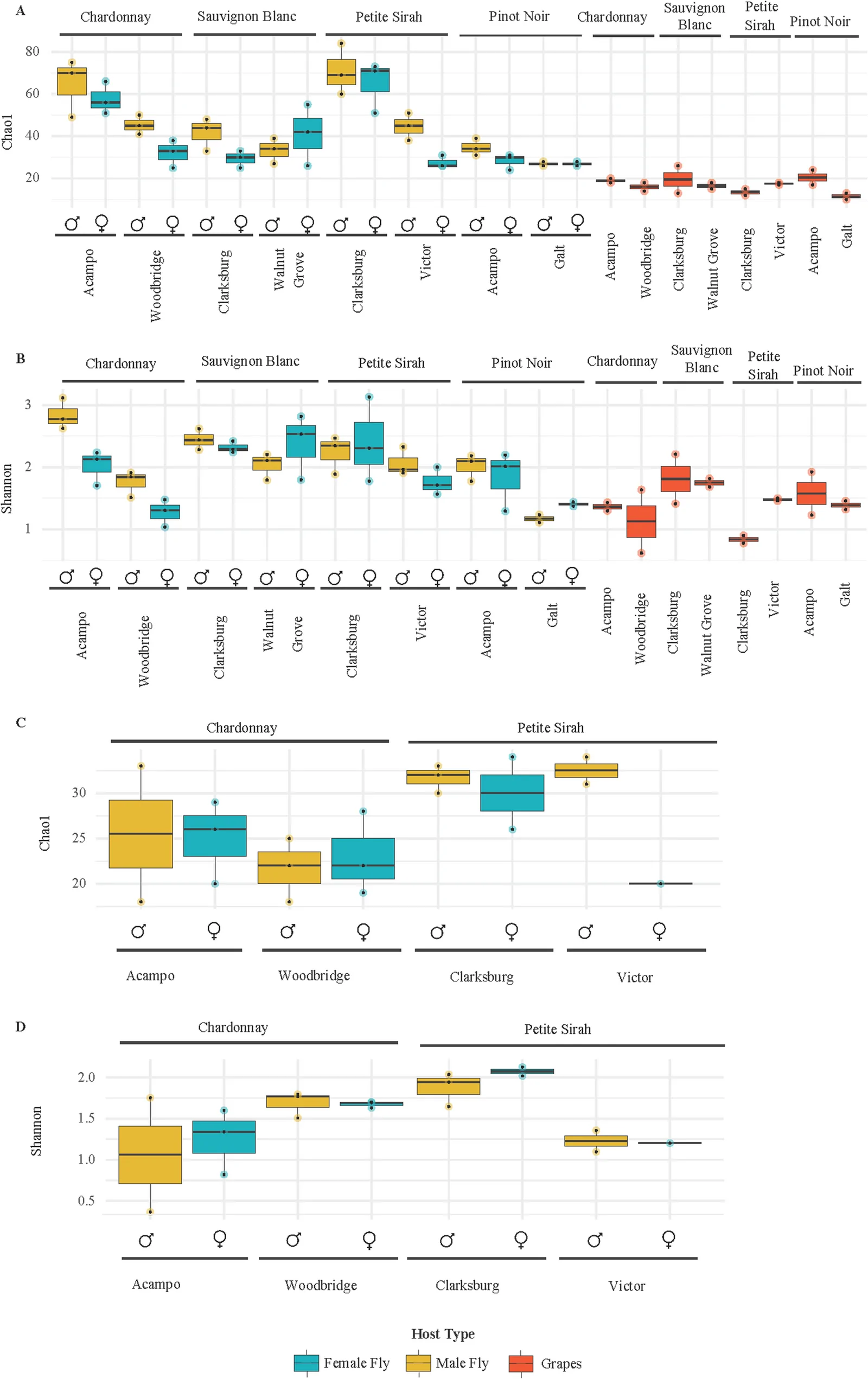
The grape microbiome was less diverse than the fly microbiome. Bacterial Chao1 richness (**A**), bacterial Shannon diversity (**B**), fungal Chao1 richness (**C**), and fungal Shannon diversity (**D**) across various host types, geographical location, and grape cultivars. Dots beyond the whiskers of the box plot represent outliers according to the 1.5 interquartile range rule

Overall, grape-associated communities were markedly less diverse than fly-associated communities, with both bacterial richness (Chao1) and Shannon diversity consistently lower in grapes than in male and female flies (Fig. 2A-B). Having established this broad compositional pattern, we used generalized linear mixed-effects models (GLMMs) to examine the effects of host type, grape cultivar, and their interaction on within-sample microbial diversity (Chao1 richness and Shannon diversity), with sampling location included as a random effect. For Chao1 richness, significant main effects were found for host type (Wald χ2 = 48.05, df = 2, *p* < 0.001), cultivar (Wald χ2 = 21.23, df = 3, *p* < 0.001), while the interaction between the host type and cultivar was not significant (Wald χ2 = 12.01, df = 6, *p* = 0.062) (Supplementary Fig. 3A, Supplementary Data 4A-B). Sampling location contributed substantially to the variation in Chao1 richness, accounting for 50.0% of the unexplained model variance (Adjusted Intraclass Correlation Coefficient, ICC) after accounting for fixed effects.

For Shannon diversity, host type (Wald χ2 = 22.55, df = 2, *p* < 0.001) and cultivar (Wald χ2 = 10.68, df = 3, *p* = 0.014) had a significant effect. Additionally, the interaction between host type and cultivar was significant (Wald χ2 = 16.64, df = 6, *p* = 0.011), indicating that the influence of grape variety on microbial diversity depends on host type (Supplementary Data 4C-D, Supplementary Fig. 3B). The random effect of location explained approximately 25.6% of the unexplained variance (Adjusted ICC) in Shannon diversity. To explicitly test the influence of SWD sex on the microbial community, independent GLMMs were fitted on SWD samples, with sex as a fixed effect. This targeted analysis confirmed no significant influence of insect sex on either Chao1 richness (Wald χ2 = 2.27, df = 1, *p* = 0.132) or Shannon diversity (Wald χ2 = 2.03, df = 1, *p* = 0.155). Together, these findings suggest that while the grapes and SWD maintain distinct alpha-diversity metrics, sampling location and cultivar play a critical role in shaping within-sample microbial diversity.

To assess the factors that influence fungal alpha diversity within SWD, generalized linear models (GLMs) were constructed using Negative Binomial and Gamma distributions for Chao1 richness and Shannon diversity, respectively. Due to sample size constraints for the fungal subset (*N* = 19), sampling location was excluded from the models to avoid rank-deficiency, thereby allowing a robust evaluation of insect sex and grape cultivar. The analysis revealed that SWD sex had no significant influence on either Chao1 richness (Wald χ2 = 0.006, df = 1, *p* = 0.940) or Shannon diversity (Wald χ2 < 0.001, df = 1, *p* = 0.987) (Fig. 2C-D, Supplementary Fig. 3C-D Supplementary Data 4E-F). Similarly, grape cultivar did not significantly affect Chao1 richness (Wald χ2 = 1.10, df = 1, *p* = 0.294) or Shannon diversity (Wald χ2 = 0.77, df = 1, *p* = 0.380) (Supplementary Data 4E-F). Furthermore, no significant interaction was observed between insect sex and cultivar for either metric (*p* > 0.05). These results confirm that, unlike the bacterial communities, fungal diversity in these SWD populations is not significantly influenced by grape cultivars.

### Microbial Composition in SWD and Grapes

In flies, the most bacterial ASVs (99.8%) were classified into three main phyla. Proteobacteria were the most abundant, accounting for 94.87%, followed by Actinobacteriota at 3%, and Firmicutes at 0.7%. Additionally, the phyla Bacteroidota and Spirochaetota appeared at lower abundances, at 0.7% and 0.6%, respectively. Conversely, grape samples were dominated mainly by Proteobacteria (99.89%), with small amounts of Actinobacteriota (0.5%) and Spirochaetota (0.5%). Notably, no members of the Bacteroidota and Firmicutes phyla were observed in the grape samples (Supplementary Fig. 4A). Among fungal ASVs, the majority were classified as Ascomycota, with an average abundance of 99.9%. A few ASVs were classified as Basidiomycota, with an average relative abundance of 0.04%. (Supplementary Fig. 4B).

**Fig. 3:**
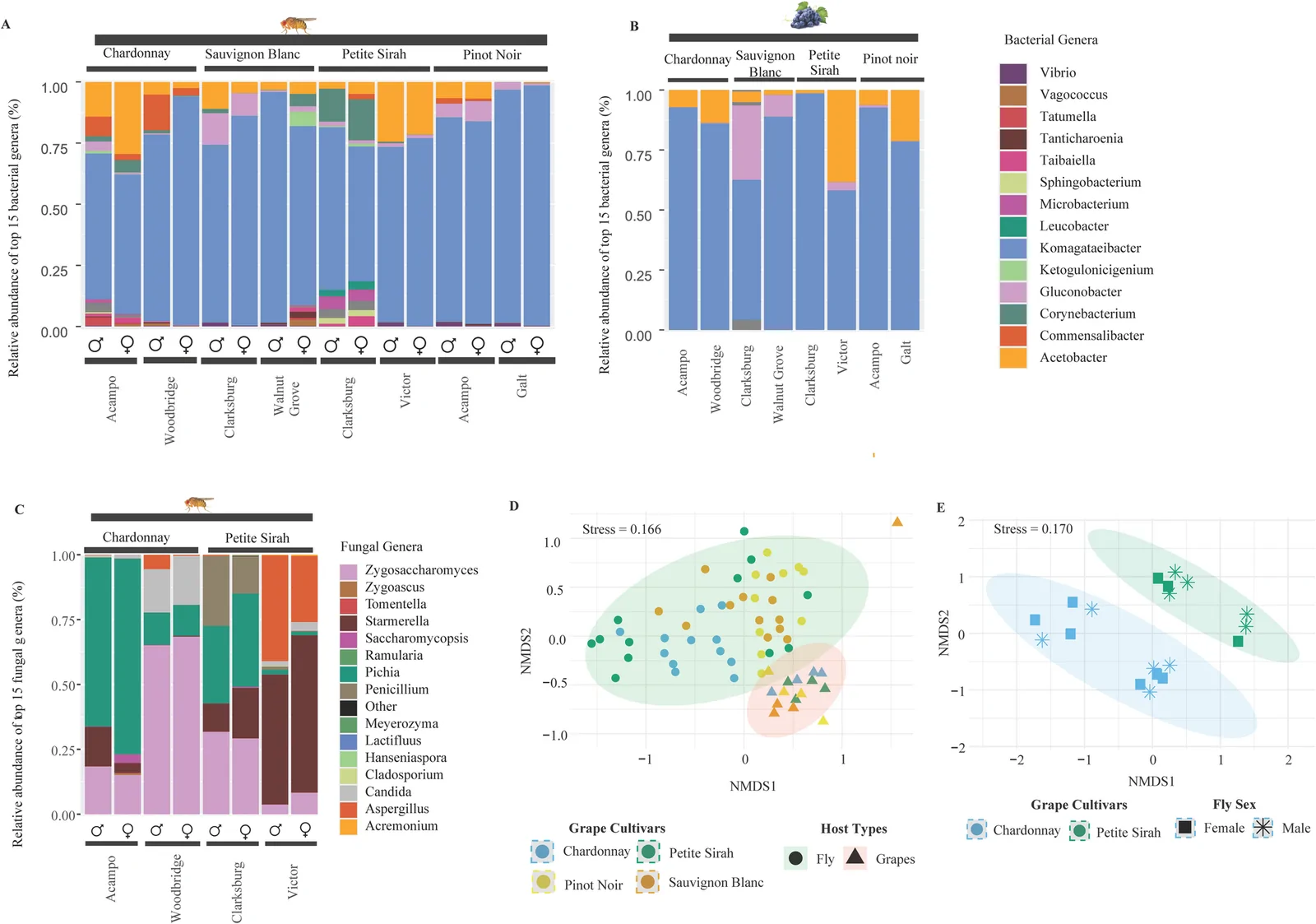
A distinct pattern of bacterial and fungal community structure is present in SWD and grapes. 16 S rRNA gene sequencing reveals relative abundances of the top 15 bacterial genera in SWD (**A**) and grapes (**B**). ITS rRNA gene sequencing reveals the relative abundances of the top 15 fungal genera in male and female SWD (**C**). Nonmetric multidimensional scaling (NMDS) ordinations based on Bray–Curtis dissimilarity for bacterial (**D**) and fungal communities (**E**). Different shapes represent the host type, while varying colors are based on the grape cultivar from which the flies and grape samples are taken

In terms of genus, *Komagataeibacter* was the most abundant genus in SWD (30.31–98.38%). Other dominant genera included *Acetobacter* (0–41.81%), *Gluconobacter* (0–17.67%), *Corynebacterium* (0–27.95%), *Commensalibacter* (0–40%), and *Ketogulonigenium* (0–14.36%). Bacterial genera, such as *Orchrobactrum*, *Microbacterium*, *Taibaiella*, *Vibrio*, *leucobacter*, *Sphinobacterium*, *Tanticharoenia*, *tatumella*, and *vagococcus* were present in lower abundances in SWD (Fig. 3A). In contrast, the bacterial genera *Komagataeibacter* (28.46–98.74%), *Acetobacter* (1–56.98%), and *Gluconobacter* (0.1–52.01%) were highly abundant in grapes. Other genera that were moderately abundant included *Methylobacterium-Methylorubrum* (0–6.89), *Bacillus* (0–4.02%), and [*Polyangium] brachysporum* group (0–1.23%). Bacterial genera belonging to *Pelomonas*, *Cutibacterium*,* Corynebacterium*,* Staphylococcus*,* Providencia*,* Clostridium*,* Tumebacillus*, and *Salinispora* were present in lower abundance in grapes (Fig. 3B). In terms of fungi, the genera *Zygosaccharomyces* (2.8–95.11%), *Pichia* (0.75–95.38%), and *Starmerella* (0.021–69.06%) were found in all the samples. On the other hand, the genera *Aspergillus* (0–53.14%), *Candida* (0–30.54%), and *Penicillium* (0–45.02%) were moderately abundant. Genera, belonging to *Saccharomycopsis*,* Xygoascus*,* Acremonium*,* Cladosporium*,* Meyerozyma*,* Tomentella*,* Hanseniaspora*,* Ramularia*, and *Lactifluss*, were present in low abundance (Fig. 3C).

### Sour Rot-Associated Microbial Genera are more Abundant in SWD Compared to Grapes

We then compared the relative abundances of bacterial and fungal genera associated with sour rot in grapes. Assessing the abundance of these microbes enables us to understand the dynamics of sour-rot pathogens within their host. Our analysis focused on the bacterial genera *Bacillus*, *Acetobacter*, *Gluconobacter*, and *Komagataeibacter*, and the fungal genera *Candida*, *Pichia*, *Aspergillus*, Saccharomyces, and *Cladosporium*, as these have been consistently implicated in the development and progression of sour rot in previous studies. Interestingly, there were no significant differences in the relative abundance of these bacterial genera across male, female, and grape samples (Supplementary Fig. 5A-D). Among fungi, we did not find significant differences in the relative abundance of the *Candida*, *Pichia*, *Aspergillus*, and *Cladosporium* genera between male and female flies (Supplementary Fig. 6A-D). However, the relative abundance of the S*accharomyces* genus was significantly higher in male flies than in female flies (Supplementary Fig. 6E). To further investigate how grape cultivar influences the abundance of sour rot-associated microbes, we compared the prevalence of key microbial genera across the different treatments. Specifically, we compared bacterial genera across four cultivars (Chardonnay, Petite Sirah, Sauvignon Blanc, and Pinot Noir) in both fly and grape samples, and fungal genera between two cultivars (Chardonnay and Petite Sirah) exclusively in the fly samples. In fly samples, the genera *Bacillus*,* Gluconobacter*, and *Komagataeibacter* exhibited significant relative abundance between cultivars (Supplementary Fig. 7). Conversely, *Gluconobacter* demonstrated only a notable difference among the grape samples, with Petite Sirah exhibiting a higher abundance than Pinot Noir and Sauvignon Blanc (Supplementary Fig. 8). Regarding fungal genera in the flies, the genus *Candida* was found to be significantly more prevalent in cultivar Chardonnay when compared to Petite Sirah and Sauvignon Blanc (Supplementary Fig. 9).

### Beta-Diversity Indicated Variation Between SWD and Grape Microbiota

To analyze compositional variations in bacterial communities across host types, grape cultivars, and sampling locations, we employed PERMANOVA to understand the extent to which each factor contributes to the structure of the SWD and grape microbiota. We utilized the Bray-Curtis distance matrix to calculate the variance explained by each factor. Subsequently, we performed a non-metric multidimensional scaling (NMDS) analysis. The results of the NMDS analysis revealed that the bacterial communities associated with SWD and grapes could be distinguished to a significant extent, with only a few exceptions between the two groups (Fig. 3D). Regarding fungal communities in SWD, we observed that the site and cultivar influenced clustering (Fig. 3E). A permutational multivariate analysis of variance on bacterial communities revealed significant effects of host type, grape cultivar, and their interaction on bacterial communities (Fig. 3D, Supplementary Data 5A). When constraining permutations by sampling location to account for spatial autocorrelation, both host type (F = 2.46, R² = 0.059, *p* = 0.001) and grape cultivar (F = 6.25, R² = 0.225, *p* = 0.001) significantly influenced bacterial community structure (Supplementary Data 5A). Notably, we did not observe any significant differences in the bacterial microbiomes between male and female flies (*p* = 0.52), whereas there were significant differences in the bacterial microbiome between male (*p* = 0.001) and female (*p* = 0.014) flies compared to grapes (Supplementary Data 5B). Geographic location emerged as a massive influencer of bacterial beta diversity. Unconstrained PERMANOVA confirmed that sampling location alone explained a substantial portion of the community variance (F = 4.75, R² = 0.341, *p* = 0.001) (Supplementary Data 5C). Furthermore, the role of location for specific cultivars explained the highest proportion of variance in the dataset (F = 5.42, R² = 0.413, *p* = 0.001) (Supplementary Data 5D). This indicates that highly localized environmental conditions strongly shape the bacterial microbiomes of both grapes and associated SWD populations.

In contrast to the bacterial communities, fungal beta diversity exhibited a distinct ecological pattern. Consistent with the fungal alpha diversity results, SWD sex had no significant impact on overall fungal community composition (F = 0.56, R² = 0.028, *p* = 0.753). However, the grape cultivar emerged as a highly significant factor, explaining over 22% of the compositional variance in the fungal communities (F = 4.49, R² = 0.222, *p* = 0.002). We did not find a significant interaction between SWD sex and cultivar (*p* = 0.977) (Supplementary Data 5E).

### Host-Associated Bacterial Networks Differ Between SWD and Grapes

To investigate differences in microbial co-occurrence patterns between the host types, we constructed separate networks for SWD and grape samples and compared their topological features using the NetCoMi package (Supplementary Fig. 10). Co-occurrence network analysis revealed differences in network topology between SWD and grape-associated bacterial communities. Within the largest connected component, the grape network had a significantly higher clustering coefficient than the fly network (0.721 vs. 0.180, *p* = 0.005), indicating that grape-associated taxa formed more locally connected groups. The grape network also had greater edge density (0.810 vs. 0.163, *p* = 0.001), natural connectivity (0.295 vs. 0.077, *p* = 0.001), vertex connectivity (4.000 vs. 1.000, *p* = 0.001), and edge connectivity (4.000 vs. 1.000, *p* = 0.001) (Supplementary Data 6). In contrast, the largest connected component size, modularity, positive edge percentage, average dissimilarity, and average path length did not differ significantly between fly and grape networks. When the whole networks were compared, the grape network had a higher clustering coefficient than the fly network (0.721 vs. 0.318, *p* = 0.019), whereas the fly network showed higher modularity than the grape network. Other whole-network properties, including the number of components, the percentage of positive edges, edge density, and natural connectivity, did not differ significantly between the two host groups (Supplementary Data 6). There was no overlap between the most central and hub taxa between the host types. Overall, these results suggest that grape-associated bacterial communities formed more locally connected and densely connected networks, whereas fly-associated bacterial communities showed greater modular separation. However, because network topology can be influenced by sample size and sparsity, these differences should be interpreted cautiously, given our smaller sample size.

### Host-Dependent Shifts in Microbial Communities

We next sought to explore how the host type influences the abundance of distinct microbial communities. An upset plot revealed that among the total of 134 ASVs, only 44 bacterial ASVs were shared across all males, females, and grape samples, belonging to the genera *Komagateibacter*, *Acetobacter*,* Gluconobacter*,* Commensalibacter*,* Cutibacterium*,* Tanticharoenia*,* Providensia*, and *Methylobacterium-Methylobrom*. Additionally, 90 ASVs were shared between male and female flies (Supplementary Fig. 11A). Notably, we did not find any ASV that was exclusively present in either grapes or male or female flies (Supplementary Fig. 11A). Furthermore, 51 fungal ASVs were detected in both male and female flies. Notably, there were five distinct fungal ASVs found exclusively in male flies, while none were observed in female flies (Supplementary Fig. 11B).

**Fig. 4:**
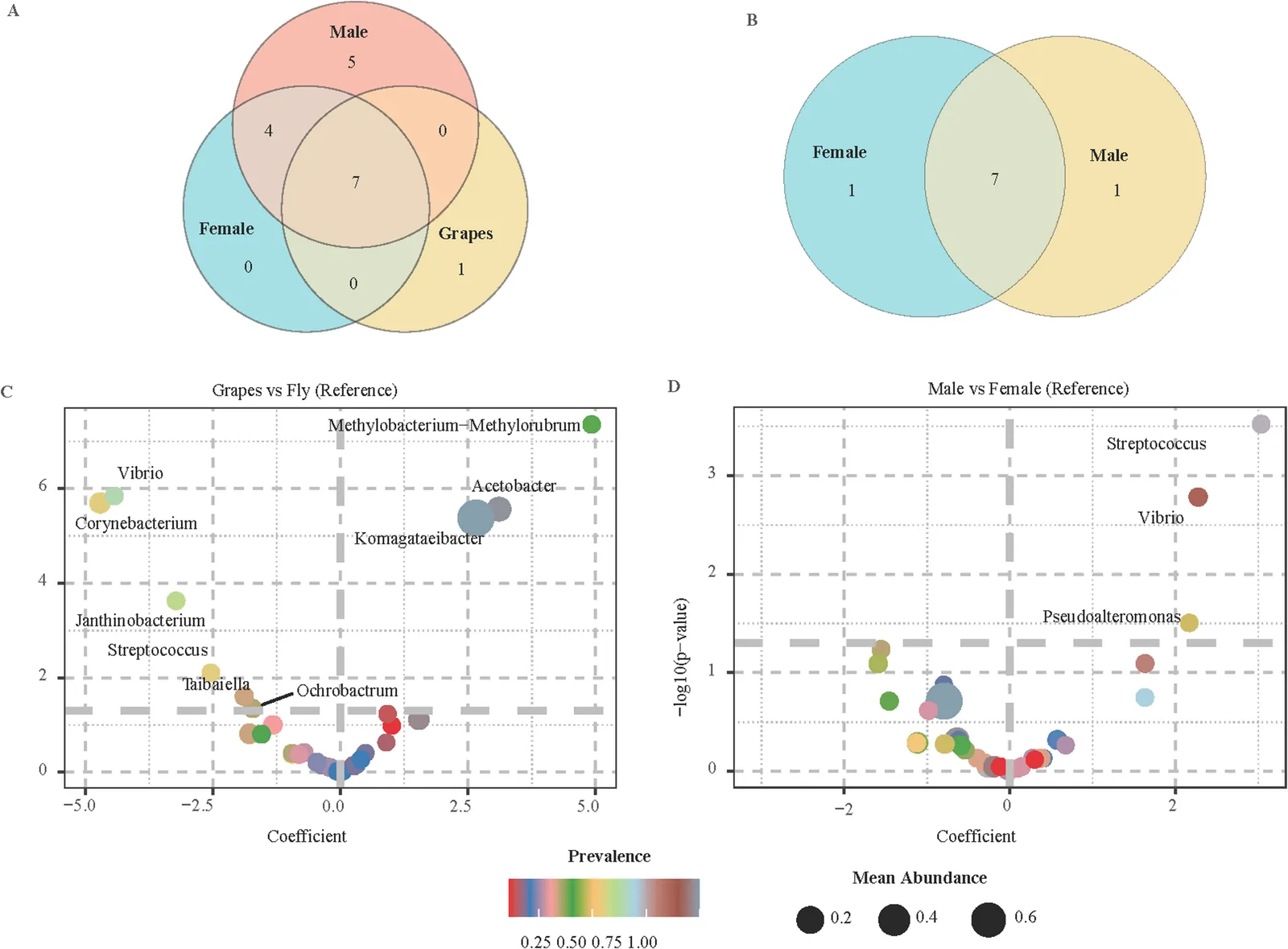
Distinct ASVs are enriched among host types, emphasizing host-driven microbiota selection. A Venn diagram illustrates the number of unique and shared core bacterial ASVs among male, female, and grape samples (**A**), and fungal ASVs between male and female fly samples (**B**). The core ASVs were defined as those with a relative abundance of at least 0.001% and were present in 70% of the samples. A volcano plot displays the estimated log2 fold difference in bacterial ASV abundance between grape and fly samples (**C**), and between male and female flies (**D**). The volcano plot shows the fold-change coefficient on the X-axis between grapes and flies. Positive values indicate higher abundance in the grapes, whereas negative values indicate higher abundance in the fly group. The Y-axis displays −log10(p-value), with values greater than one indicating statistically significant differences between groups

To investigate the proportion of shared taxa among samples from different sites, we analyzed the core microbial community, comprising taxa present in at least 0.01% of samples and detected in at least 70% of samples. Seven bacterial core taxa belonging to the genera *Komagataeibacter*, *Acetobacter*, and *Gluconobacter* were observed in both the fly and grape samples (Fig. 4A). In contrast, male flies exhibited distinct ASVs belonging to the genera *Corynebacterium*, *Cutibacterium*, and *Streptococcus*, while female flies lacked distinct core ASVs (Fig. 4A). Analyzing fungal ASVs between male and female flies revealed seven core fungal ASVs belonging to the genera *Aspergillus*, *Starmerella*, *Zygosaccharomyces*, and *Pichia*, which were found in both sexes (Fig. 4B).

To identify host-specific microbial signatures, we used linear models for differential abundance analysis (LinDA) to detect ASVs that were differentially abundant across flies and grapes, and between male and female flies. Compared with flies, bacterial communities in grapes showed significant depletion of several genera, including *Corynebacterium*, *Vibrio*, *Taibaiella*, *Orchobactrum*, *Janthinobacterium*, and *Streptococcus*. In contrast, *Methylobacterium-Methlorubrum*, *Acetobacter*, and *Komagataeibacter* were enriched in grapes compared to flies (Fig. 4C). Feature-level differential abundance analysis between male and felame SWD revealed that male flies were significantly enriched in *Vibrio*, *Pseudoalteromonas*, and *Streptococcus*, whereas no taxa were significantly enriched in female flies. (Fig. 4D). In terms of fungal genera across male and female flies, we identified *Ramularia* and *Cladosporium* as enriched in male flies, while no differentially significant genera were found in female flies (Supplementary Fig. 11C).

### Microbial Communities in Grapes have Higher Community Dissimilarity with Geographical Distance than in SWD

**Fig. 5:**
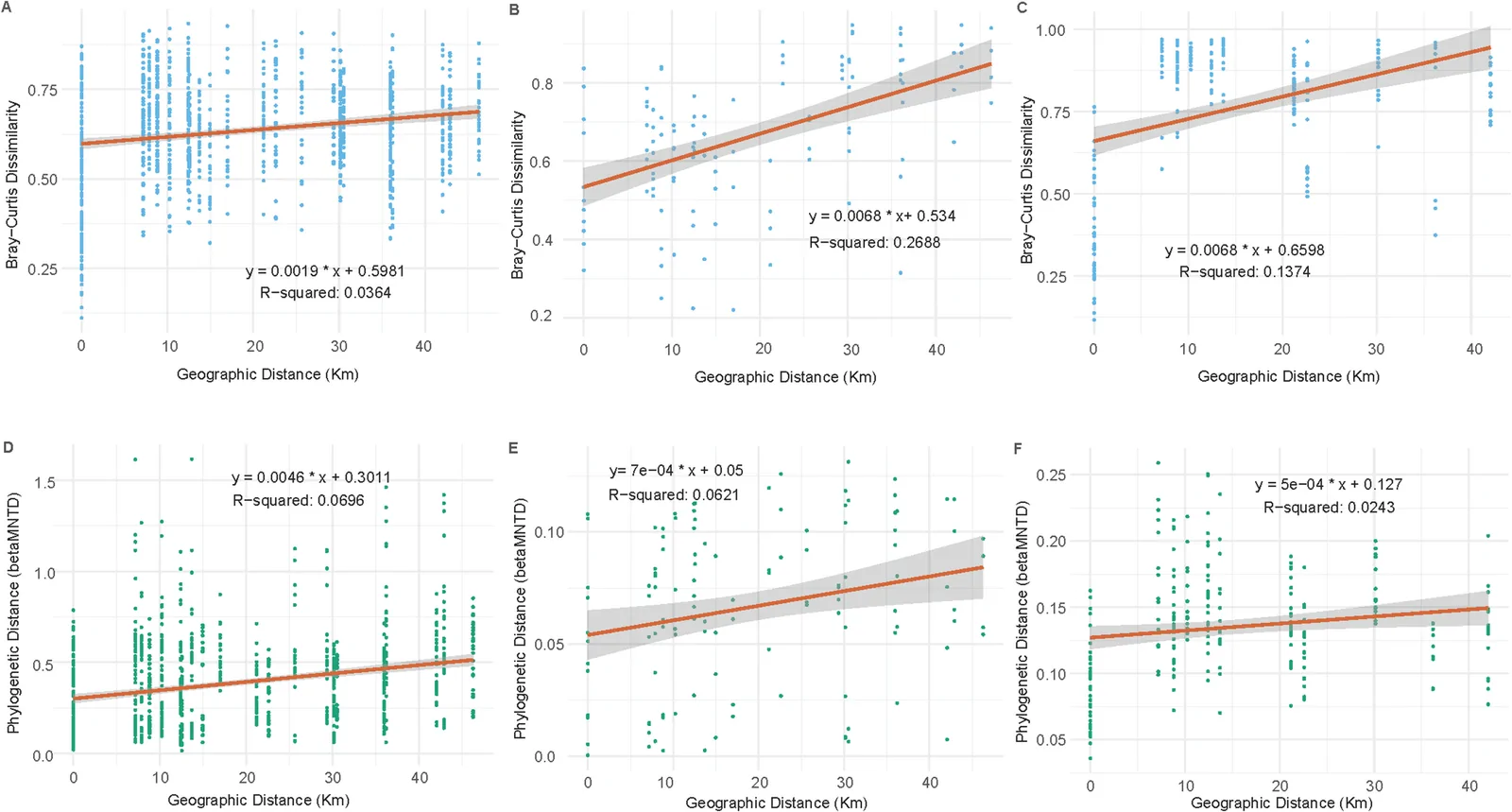
Biogeographical patterns and phylogenetic diversity of bacterial and fungal microbiomes in SWD and grapes. Distance decay relationship of microbial community dissimilarity in male and female flies and grapes as assessed by linear regression between bacterial community based on Bray-Curtis dissimilarity in the fly (**A**), grapes (**B**), and fungal communities in the fly (**C**). Phylogenetic distance calculated using betaMNTD (beta Mean Nearest Taxon Distance), and distance decay analyses performed across flies (**D**), grapes (**E**), and fungal communities in flies (**F**). Statistical significance of all distance-decay correlations was assessed using matrix-based Mantel tests to account for pairwise non-independence. The red lines represent fitted linear models with shaded areas indicating 95% confidence intervals, to visualize the spatial trends

We analyzed the distance decay of total bacterial and fungal community dissimilarity to understand how geographic distance between sampling locations influences differences in bacterial and fungal communities. Bray–Curtis dissimilarity was positively associated with geographic distance in grapes (Mantel *r* = 0.520, *p* = 0.004), indicating evidence of spatial turnover in grape-associated bacterial communities. However, a weaker association was observed for fly-associated bacterial communities (Mantel *r* = 0.140, *p* = 0.022). These findings suggest that spatially closer samples exhibited more similar community structures in grapes and a stronger decay of community dissimilarity with geographic distance, with higher spatial species turnover than flies (Fig. 5A and B). We employed phylogenetic distances derived from beta-mean nearest taxon distance (β-MNTD) matrices to unravel the phylogenetic structure of communities and assess variation in community similarity across geographic distances. For bacteria, we did not find a significant correlation between phylogenetic distance and geographical location in both SWD (Fig. 5D) and grapes (Mantel *r* = 0.173, *p* = 0.152) (Fig. 5E). For SWD fungal communities, we observed a weak but significant positive correlation between Bray-Curtis dissimilarity (mantel *r* = 0.2391, *p* = 0.007) (Fig. 5C) and phylogenetic distance (mantel *r* = 0.2055, *P* = 0.017) (Fig. 5F). Overall, these results indicate that grape-associated bacterial communities experience spatial turnover influenced by environmental factors, whereas fly-associated bacterial communities remain highly consistent across locations, likely shaped by host filtering or dispersal. While fly-associated fungal communities demonstrate statistically significant distance-decay patterns, the modest effect sizes suggest a limited regional divergence. Notably, because these spatial inferences are drawn from a restricted number of sampling sites (seven total locations for bacteria; four for fungi).

### Stochastic Processes Primarily Shape Microbial Community Assembly in SWD, While Non-stochastic Processes have a Minor Influence on Grape Assembly

Classification of taxa as abundant, intermediate, and rare was performed using an unsupervised machine learning approach with the ulrb package in R. The rank abundance curve across samples aligned with the classical RAC, exhibiting a long tail of rare ASVs, an intermediate region of undetermined ASVs, and a few bacterial and fungal ASVs with exceptionally high abundance (Supplementary Fig. 13). Our subsequent classification of ASVs across the population revealed that only a single bacterial ASV, belonging to the genus *Komagataeibacter*, was classified as abundant. In contrast, 106 ASVs were classified as rare, and 7 as intermediate taxa in flies. Regarding grapes, 1 ASV from the genus *Komagataeibacter* was classified as abundant, 5 as intermediate, and 13 as rare taxa. In terms of fungal ASVs in the fly, 9 ASVs were found as intermediate, and 40 ASVs were classified as rare, with no abundant ASVs identified.

**Fig. 6:**
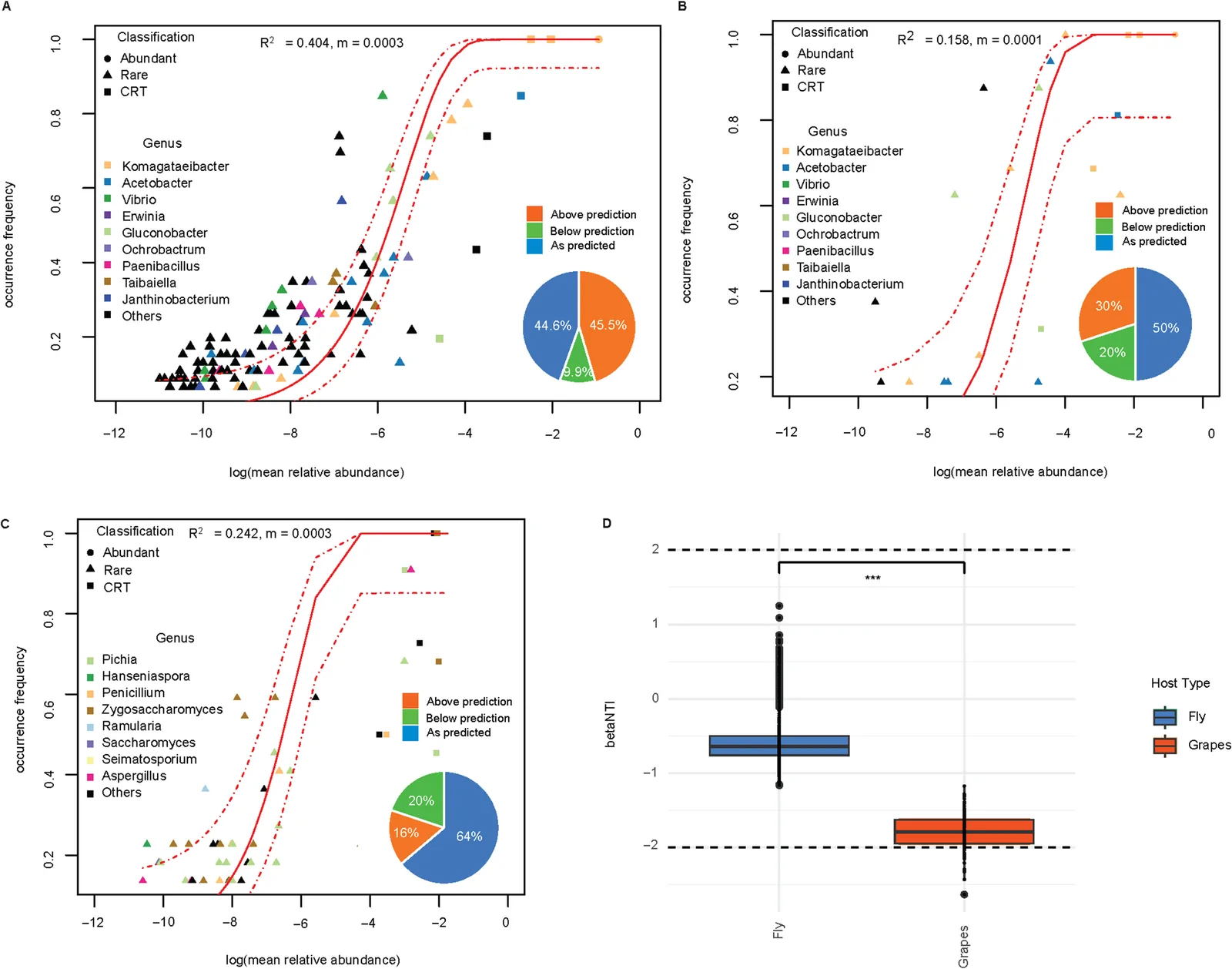
Stochastic processes dominate SWD microbiome assembly. Sloan neutral model prediction of bacterial microbial communities in fly (**A**), grapes (**B**), and fungal microbial communities in fly (**C**). ASVs are represented as data points and colored by genus. R^2^ values (measurement of fit to the neutral assembly process) and *m* values (estimated migration rate) are indicated for each prediction. The pie chart represents the proportion of ASVs predicted as, above or below the confidence interval according to their Sloan neutral model prediction. Each dot represents a taxon, and the solid line is the best-fitting neutral community expectation. The dashed lines and gray area depict the 95% confidence bands. (**D**) Box plot of beta nearest taxon index (βNTI) showing community assembly processes of bacterial community across host types. A βNTI value greater than 2 or less than − 2 indicates the predominance of deterministic (variable selection + homogeneous selection) processes, while values between − 2 and 2 suggest stochastic processes (dispersal limitation + homogenizing dispersal + ecological drift)

To explore the relative importance of neutral and non-neutral processes in shaping bacterial and fungal microbial community assemblies in flies and grapes, we fitted bacterial and fungal ASVs to the Sloan neutral model to estimate each ASV’s occurrence and relative abundance. We observed that the Sloan neutral model fit the neutral community for bacterial communities in flies (R^2^ = 0.404) (Fig. 6A) better than for bacterial communities in grapes (R^2^ = 0.158) (Fig. 6B) and for fungal microbial communities in flies (R^2^ = 0.242) (Fig. 6C). This suggests a more natural balance between dispersal and selection in the fly ecosystem. The higher explanation rate of the neutral community model for bacterial communities in flies than in grapes might suggest that stochastic processes played a more significant role in bacterial community assembly in flies than in grapes. To investigate whether abundant or rare microbial communities follow the neutral microbial assembly process, we analyzed all rare bacterial and fungal taxa in flies and grapes.

In the fly, the Neutral model identified 53 ASVs exhibiting signs of positive selection, characterized by higher-than-expected prevalence and the presence of all rare taxa (Supplementary Data 7A). Notably, only 12 ASVs fell below the predicted threshold, predominantly belonging to rare and intermediate genera such as *Komagataeibacter*,* Acetobacter*, *Gluconobacter*, *Corynebacterium*, and *Commensalibacter* (Supplementary Data 7A). Regarding grapes, 6 bacterial ASVs belonging to the genera *Escherichia*,* Glucanobacter*,* Methylobacterium*, and *Komagataeibacter* were found above the neutral prediction threshold, which includes abundant, intermediate, and rare ASVs (Supplementary Data 7B). We had 5 ASVs below the neutral prediction threshold (Supplementary Data 7B). Among the fugal genera associated with flies, 8 ASVs belonging to the genera *Zygosaccharomyces*,* Saccharomyces*,* Hanseniaspora*, and *Ramularia* were explicitly found above the neutral model, while 121 ASVs belonging to the genera *Zygosaccharomyces*,* Saccharomyces*,* Habseniaspora*,* Pichia*, and *Ramularia* were found below the prediction threshold (Supplementary Data 7C). This suggests that few rare and intermediate microbial communities are predominantly selected for or against in nature, while most rare microbial communities occur by chance. Furthermore, the neutral model analysis revealed low migration rates (m = 0.0001–0.0003) across all sample types, indicating strong dispersal limitations in fly and grape ecosystems.

As the null model revealed that stochastic processes predominated in bacterial community assembly in flies compared to grapes, the beta-nearest taxon index (βNTI) was used to assess the relative impact of deterministic and stochastic processes on bacterial community assembly. βNTI > 2, < −2, and between − 2 and 2 are interpreted as variable selection of deterministic processes, homogeneous selection of deterministic processes, and stochastic processes, respectively. The difference analysis of βNTI values between treatment types revealed significant differences between grapes and SWD (Wilcox-Rank, *p* < 0.001) (Fig. 6D). Notably, βNTI values for SWD were within the range of −2 to 2, suggesting that the assembly dynamics of bacterial communities in SWD are predominantly governed by stochastic processes. Regarding grapes, 12% of the βNTI values were less than − 2, while approximately 88% were between − 2 and 2. This finding suggests that deterministic processes (homogeneous selection) play a crucial role in the assembly of the grape bacterial microbiome, alongside stochastic processes (Fig. 6D).

Regarding the fungal microbiome in male and female SWD flies, we found no significant difference in βNTI values (*p* = 0.658 (Supplementary Fig. 14). Approximately 90% of the βNTI values were between − 2 and 2, while about 10% were below − 2. This indicates that the assembly process of the SWD fungal microbiome communities is predominantly governed by stochastic processes, with some influence of deterministic processes.

## Discussion

Understanding the assembly processes of the fly and its plant host-associated microbial communities is crucial for understanding interactions and succession under diverse environmental conditions, thereby enabling effective pest management. In this study, we used high-throughput sequencing of 16 S and ITS rRNA genes for community assembly analysis, co-occurrence network analysis, and differential abundance to examine the abundance of abundant, intermediate, and rare bacterial and fungal taxa associated with sour rot in grape and SWD flies from four distinct cultivars and seven different locations in the Northern San Joaquin Valley of California, USA.

Our study showed that female SWD were significantly larger than male SWD (Fig. 1A), consistent with previous findings [81]. A DNA-based method for estimating microbiota density has been widely used in microbiome studies [69, 82, 83]. Microbiota density, measured as micrograms of DNA per milligram of sample, was lower in male flies than in female flies. Additionally, the microbiota density in grapes was also significantly lower than in flies (Fig. 1B), indicating that flies contain more microbial biomass per unit of body weight than grapes. The phyllosphere generally harbors a comparatively low microbial load, likely due to the harsh and inhospitable conditions of its microenvironment [84, 85]. The reduced microbiota density observed in grape samples may reflect this inherently lower microbial abundance in the phyllosphere, as well as the effects of sour rot infection, which could further diminish both microbial density and richness. Various studies have also suggested that dysbiosis is often associated with reduced microbial richness and disease symptoms in the human gut [69, 86, 87] and plants [88–90]. While previous studies on grape sour rot have reported conflicting patterns of microbial richness between infected and healthy grapes [91, 92], our work examined only sour-rot–affected samples and therefore could not make such comparisons. The lower microbiota density observed in grape samples appears to contribute to their reduced microbial diversity and richness compared to fly samples (Fig. 2). This pattern suggests that higher microbiota density may promote greater microbial diversity and richness. Biases in microbial composition analyses can arise from differences in DNA isolation techniques and commercial extraction kits [93–95]. Accordingly, variation in extraction methodology, an uncontrolled factor in this study, may partly explain the observed disparity in microbial density between grapes and flies. The comparison of bacterial communities showed that the SWD had significantly different bacterial communities than those of grapes, with only a small fraction of the total microbes shared between the fly and grapes (Fig. 3D). SWD are highly polyphagous and target a wide range of wild and ornamental fruits [96]. The fly’s ability to feed on a wide variety of host plants and overwinter in different forest vegetation, even without grapes, might explain the variation in its microbiome composition [97, 98]. We hypothesized that the diverse host populations provide more variable environments for microbes, enabling a greater diversity of microbes to persist or supporting greater microbiome diversity through a higher number of functionally important host genotype-microbial taxon-specific interactions. Previous studies have also indicated that the microbiome community in various insects is primarily derived from the host plant [99, 100]. Moreover, bacterial communities coexisting within the insect body are acquired from multiple sources, including environmental acquisition and vertical transmission [101, 102]. These bacteria persist in the flies due to frequent reintroductions via food sources. Consequently, these cycles of reintroductions, transmission, and colonization of microbial communities in a fly could contribute to the persistence and distribution of these microbial groups.

Sour rot of grapes is a disease complex involving Drosophila flies, yeast, and acetic acid bacteria. A comprehensive review of the literature on sour rot and its associated pathogens reveals that the predominant fungal genera implicated in the disease includes *Candida*, *Pichia*, *Saccharomyces*, and *Aspergillus*. Among bacteria, acetic acid bacteria (AAB) are consistently identified as key contributors, with dominant genera including *Acetobacter*, *Gluconobacter*, *Bacillus*, and *Komagataeibacter* [36, 92]. We compared the relative abundances of these fungal and bacterial genera between SWD and grapes and found no significant differences. In Drosophila, microbiota assembly is influenced by multiple processes, including vertical transmission and environmental acquisition However, the constraints of our experimental design prevented us from directly inferring microbial transfer between flies and grapes through compositional source tracking, as we lacked the environmental controls and reference samples required for such analyses. Consequently, further experimental validation is necessary to substantiate this potential source–sink relationship. Further experimental validation is necessary to substantiate this potential source-pathogen relationship. In terms of cultivar, Chardonnay had the highest abundance of the sour rot pathogen, followed by Petite Sirah, indicating a selective influence of cultivars on its abundance. These cultivars are characterized by thin-skinned berries and tight clusters. Grape cultivars with this combination of morphological features are particularly more prone to sour rot [37].

Studies on the Drosophila microbiome have revealed that the microbiome significantly influences the fruit fly’s physiology and biological functions. However, these microbial communities are subject to change in response to various factors, such as geographic location [103, 104], diet [105, 106], and host crop [104, 107]. Despite the ever-changing nature of the microbiome, a stable subset of microorganisms, known as the core microbiota, persists. These core microbiotas possess unique characteristics or functions within the ecosystem and may have coevolved with the host [108]. Our study found that *Komagataeibacter*, *Acetobacter*, and *Gluconobacter* are core bacterial genera present in both grape and SWD samples. These findings align with observations from various surveys of wild *D. suzukii* [104, 106, 109, 110]. These acetic acid bacteria possess membrane‑bound dehydrogenases that oxidize ethanol to acetic acid, a process responsible for wine spoilage. They are also notable for their tolerance to low pH and their role in modulating innate immunity and insulin signaling [111, 112], thereby conferring beneficial physiological effects on the fly host. Furthermore, their capacity to employ quorum sensing to regulate biofilm formation [113], upregulate stress-response chaperones [114], and activate the type II toxin–antitoxin system [115] may enhance their resilience under diverse stress conditions within the host environment. In terms of fungi in SWD, ASVs belonging to the genera *Aspergillus*, *Starmerella*, *Zygosaccharomyces*, and *Pichia* were found in both male and female flies. As previously observed, female flies tend to have higher abundances of yeast genera than male flies [116]. The yeast genera *Starmerella*, *Zygosaccharomyces*, and *Pichia* are well known for their fermentative activity and also play essential roles in Drosophila physiology, performance, and behavior [117]. Their ability to form a chitosan-layered spore wall [118], coupled with the presence of stress-protective cellular factors [119], and traits such as auto-aggregation and adhesion [120], may enhance their capacity to withstand the hostile environment. Our analysis revealed that bacterial diversity in SWD and grapes is influenced by host type, cultivar, and location, reflecting the combined effects of host-microbe interactions and environmental conditions. Although we found no significant differences in microbial communities between male and female flies, our study did not account for the potential influence of fly genotype on this variation. A survey of SWD across the continental US revealed distinct population structures between Western and Eastern regions, but no genetic differentiation across latitudes [121]. These genotypic patterns along the longitudinal axis may also influence microbiota composition, suggesting a dynamic interaction between host genetics and local ecology that could be further examined through extensive longitudinal studies.

Exploring microbial communities, particularly their biogeographic distribution and community assembly processes, sheds light on the interactions and succession of microbes under diverse environmental conditions [122]. Comparing distance-decay patterns, microbial community dissimilarity increased more rapidly with geographic distance in grapes than in flies, indicating a stronger spatial turnover in grape-associated microbiota. These might be attributed to the mobile nature of SWD, while grapes are more prone to selection. *D. melanogaster*, a close relative of SWD, can disperse up to 12 km in still air and even farther with moderate wind conditions [123], which likely contributes to the weaker distance decay observed in fly-associated microbial communities. This high dispersal potential may facilitate microbial homogenization across geographic regions, resulting in reduced spatial turnover compared to the more localized grape microbiota.

Once microbes are transmitted to the host, various deterministic and stochastic processes influence the assembly and maintenance of the microbial community [124, 125]. Neutral community model analysis revealed that neutral processes influence microbial community structure in flies more than in grapes (Fig. 6). In natural environments, Drosophila acquire microbes from their diet and surrounding habitat, collectively known as the regional species pool, resulting in rapid, reproducible shifts in their microbiomes. However, the fundamental processes driving microbial community assembly in wild Drosophila remain largely unexplored. Our results showed that ecological drift and passive dispersal primarily influence the microbial abundance in SWD. In contrast, homogeneous selection also plays a significant role in shaping microbial communities associated with grapes, indicating that microbial community assembly processes are not conserved across ecosystems. Notably, similar patterns of neutral processes shaping microbiome composition have been previously reported in *Drosophila* species [126–128], further supporting the generality of these mechanisms in insect-microbe interactions. Previous studies on microbial community assembly in diverse wild insect taxa indicate that host identity can shape microbiome composition; however, stochastic processes often exert a stronger influence than deterministic filtering [129–132]. Mobility of the insects across diverse environments and food sources increases their exposure to variable microbial pools, potentially favoring stochastic over deterministic processes in microbiome assembly. The extent of this influence, however, remains to be tested. Low-abundance microorganisms, often referred to as the rare biosphere, play a crucial role in ecosystem resistance and resilience. Microbial communities were classified into abundance categories: rare, intermediate, and abundant. ASVs that fell outside the neutral model’s confidence intervals, indicating host-driven selection, were predominantly rare taxa, suggesting that these low-abundance bacteria play a critical role in maintaining the stability and functionality of the wild SWD and grape microbiome, a pattern consistent with findings from other ecosystems [21, 133, 134]. Such taxa may act as keystone species within these microbial networks, a possibility that warrants further investigation. One limitation of this study is the predominance of rare taxa within the microbial communities, which may have disproportionately influenced the observed patterns of community assembly. As a result, the role of abundant taxa may be underrepresented, and further analyses incorporating larger, more diverse datasets would be valuable to validate these findings and assess the broader applicability of rare-taxa-influenced assembly processes.

In summary, this study comprehensively examines the bacterial and fungal communities associated with SWD and sour-rot-associated grapes. Our findings reveal that the flies harbor a diverse microbial community, in contrast to the phyllosphere microbial communities found on grapes. Additionally, we propose that the insect microbiome is shaped by complex interactions among host-microbe dynamics, geographic location, and environmental factors, resulting in distinct compositions across hosts and locations. Our analysis of the assembly processes suggests that both selective and neutral mechanisms contribute to the formation of abundant and rare taxa, although their respective impacts on community composition differ. These findings provide a foundation for microbiome-informed strategies in integrated disease management. The identification of rare yet influential microbial taxa associated with sour rot suggests potential targets for microbial manipulation to mitigate disease impact. By incorporating microbiome dynamics into pest and disease control frameworks, future approaches could enhance the resilience and health of grape crops through more sustainable and ecologically informed practices.

## Supplementary Information

Below is the link to the electronic supplementary material.

Supplementary Material 1 (XLSX 15.6 KB)

Supplementary Material 2 (XLSX 13.8 KB)

Supplementary Material 3 (XLSX 11.6 KB)

Supplementary Material 4 (XLSX 14.6 KB)

Supplementary Material 5 (XLSX 57.3 KB)

Supplementary Material 6 (XLSX 31.8 KB)

Supplementary Material 7 (XLSX 11.2 KB)

Supplementary Material 8 (DOCX 1.70 MB)

## Data Availability

Raw sequencing data can be accessed in the National Center for Biotechnology Information (NCBI) Sequence Read Archive (SRA) under the BioProject accession number PRJNA1246986. All the data and code associated with this study are available in the following GitHub repository (https://github.com/DavidKang-USDA/Sour-rot-microbiome-assembly).
