# Supplementary material for: Rare Taxa and Stochastic Drift Drive the Microbiome Assembly of the Invasive Pest Drosophila suzukii, While Host Filtering Structures the Grape Sour Rot Community
Source: Microb Ecol. 2026 Jun 26;89:173. doi: 10.1007/s00248-026-02819-x (PMC13562485; doi:10.1007/s00248-026-02819-x)
Supplement: Supplementary file 1 [file 248_2026_2819_MOESM8_ESM.docx]

**Rare taxa and stochastic drift drive the microbiome assembly of the invasive pest *Drosophila suzukii*, while host filtering structures the grape sour rot community**

Rishi Bhandari^1^, Zoe Wills^1^, Jackie Harris^2^, Marnelle Salie^2^, Nana Y. D. Ankrah^3^, Adam Chun-Nin Wong^4^, Katharine Eastman^1^, Joseph Ringbauer Jr^1^, R. Keith Striegler^2^, and David S. Kang^1^

^1^Biological Control of Insects Research Laboratory, US Department of Agriculture, Agricultural Research Service, Columbia, Missouri, USA

^2^E & J. Gallo Winery, Acampo, California, USA

^3^Biological Science Department, State University of New York Plattsburgh, Plattsburgh, New York, USA

^4^Department of Entomology and Nematology, University of Florida, Gainesville, Florida, USA

**Supplementary Figures**

**Supplementary Figure 1: Vineyard sampling locations in California.** Map of California indicating the geographic locations of the vineyards where samples were collected.

# **Supplementary Figure 2: Rarefaction curve for bacterial and fungal communities.** The species diversity rarefaction curve indicates adequate sequencing coverage for both bacterial (A) and fungal (B) communities.

**Supplementary Figure 3: Alpha diversity across host types.** Chao1 richness (A) and Shannon diversity (B) of bacterial communities across different host types. Chao1 richness (C) and Shannon diversity (D) of fungal communities in male and female SWD.

**Supplementary Figure 4: Phylum-level taxonomic composition across treatment groups.** Relative abundance of bacteria (A) and fungal (B) phyla across different treatment types.

**Supplementary Figure 5: Relative abundance of bacterial genera associated with sour rot in SWD and grapes.** Box plot illustrating the relative abundance (A-D) of bacterial genera linked to sour rot in male and female SWD flies and grape samples.

**Supplementary Figure 6: Relative abundance of fungal genera associated with sour rot in SWD.** Box plots showing the relative (A-D) abundance of the fungal genera associated with sour rot in male and female SWD flies.

**Supplementary Figure 7: Relative abundance of bacterial genera associated with sour rot in SWD across various grape cultivars.** Box plot illustrating the relative (A-D) abundance of bacterial genera associated with sour rot detected in fly samples from various grape cultivars.

**Supplementary Figure 8: Relative abundance of bacterial genera associated with sour rot in grape samples across various grape cultivars.** Box plot illustrates the relative (A-D) abundance of bacterial genera associated with sour rot detected in grape samples from various grape cultivars.

**Supplementary Figure 9: Relative abundance of fungal genera associated with sour rot in SWD across various grape cultivars.** Box plot showing the relative (A-D) abundance of the fungal genera associated with sour rot in fly samples across various grape cultivars.

**Supplementary Figure 10: Bacterial co-occurrence networks in SWD and grape samples.** Comparison of bacterial association networks in fly and grape samples. Hub taxa are highlighted in bold text. Node color indicates the cluster identified by greedy modularity optimization. Red edges indicate negative associations, while blue edges indicate positive ones.

**Supplementary Figure 11: Distributions of amplicon sequence variants (ASVs) across different treatment groups**. Total number of bacterial ASVs in grape, male, and female fly samples (A), and fungal ASVs in male and female flies (B). Horizontal bars represent the set size for each group, indicating the number of OTUs unique to each, while dark circles show shared OTUs between samples. Connecting lines illustrate OTUs common to multiple groups. A volcano plot displays the estimated log2 fold difference in fungal ASV abundance between male and female flies, with positive values indicating higher abundance in males and negative values indicating higher abundance in females.

**Supplementary Figure 12: Classification of ASVs into the abundant class across all samples.** In each sample, phylogenetic units are arranged on the x-axis from most to least abundant, while the y-axis indicates their abundance scores. Silhouette scores for each unit across all samples are shown on the right. The ASVs are color-coded according to the default classification: rare, undetermined, and abundant.

**Supplementary Figure 13: Distribution of standardized phylogenetic turnover based on the beta nearest taxon index (βNTI) of fungal microbial communities in male and female flies.** βNTI values were compared between male and female flies using the Wilcoxon rank sum test (ns, *p* > 0.05). The horizontal dotted line (above +2 or below −2 is statistically significant and shows the 95% confidence intervals around the expectation under neutral community assembly.

**Supplementary Data**

**Supplementary Data 1: Sample metadata associated with the study.** This file contains detailed metadata for all samples included in the study, including sample identifiers, collection dates, experimental conditions, and relevant descriptive attributes. These metadata were used for downstream analyses and interpretation of results.

**Supplementary Data 2: Total read counts across each sample associated with the study.** This file contains the total sequencing read counts for bacterial and fungal communities across all samples included in the study. Each row corresponds to a sample, and the columns provide the number of bacterial reads, fungal reads, and the combined total. These counts were used for quality assessment and normalization in downstream analyses.

**Supplementary Data 3: Taxonomic classification and associated read counts*.*** This file contains the taxonomic classification of sequences obtained in the study, along with the corresponding read counts for each taxon. Taxa are organized hierarchically (e.g., phylum, class, order, family, genus) and include both bacterial and fungal groups. These data were used for community composition analysis and diversity assessments.

**Supplementary Data 4: Statistical tests for bacterial and fungal alpha diversity*.*** This file contains the results of ANOVA tests performed to assess differences in bacterial and fungal alpha diversity across experimental groups. Post hoc pairwise comparisons (e.g., Tukey’s HSD) are included to identify significant differences between specific group pairs. The file reports test statistics, degrees of freedom, p-values, and adjusted significance levels.

**Supplementary Data 5: PERMANOVA results for bacterial and fungal beta diversity.** This file contains the results of PERMANOVA (Permutational Multivariate Analysis of Variance) tests assessing differences in bacterial and fungal beta diversity across various experimental factors. The output includes pseudo-F statistics, degrees of freedom, p-values, and R² values for each factor tested. These analyses were performed on distance matrices derived from community composition data to evaluate the influence of experimental variables on microbial community structure.

**Supplementary Data 6: Topological features for bacterial network analysis.** This file contains the computed topological properties of bacterial co-occurrence networks derived from the study data. Metrics include node degree, betweenness centrality, closeness centrality, clustering coefficient, and modularity scores for each network. These features were used to assess network structure, identify key taxa, and evaluate connectivity patterns within bacterial communities.

**Supplementary Data 7: Neutral model predictions for ASVs in different hosts.** This file contains the results of neutral community model predictions for bacterial and fungal amplicon sequence variants (ASVs) across different host organisms. The dataset includes observed and predicted occurrence frequencies, model fit parameters, and classification of ASVs as neutral or non-neutral. These predictions were used to evaluate the role of stochastic processes in shaping microbial communities within each host type.
